# Neural Stem Cells of the Subventricular Zone as the Origin of Human Glioblastoma Stem Cells. Therapeutic Implications

**DOI:** 10.3389/fonc.2019.00779

**Published:** 2019-08-20

**Authors:** Esperanza R. Matarredona, Angel M. Pastor

**Affiliations:** Departamento de Fisiología, Facultad de Biología, Universidad de Sevilla, Seville, Spain

**Keywords:** glioblastoma stem cells, tumor microenvironment, adult neurogenesis, neural stem cells, subventricular zone, driver mutations

## Abstract

Human glioblastoma is the most aggressive type of primary malignant brain tumors. Standard treatment includes surgical resection followed by radiation and chemotherapy but it only provides short-term benefits and the prognosis of these brain tumors is still very poor. Glioblastomas contain a population of glioma stem cells (GSCs), with self-renewal ability, which are partly responsible for the tumor resistance to therapy and for the tumor recurrence after treatment. The human adult subventricular zone contains astrocyte-like neural stem cells (NSCs) that are probably reminiscent of the radial glia present in embryonic brain development. There are numerous molecules involved in the biology of subventricular zone NSCs that are also instrumental in glioblastoma development. These include cytoskeletal proteins, telomerase, tumor suppressor proteins, transcription factors, and growth factors. Interestingly, genes encoding these molecules are frequently mutated in glioblastoma cells. Indeed, it has been recently shown that NSCs in the subventricular zone are a potential cell of origin that contains the driver mutations of human glioblastoma. In this review we will describe common features between GSCs and subventricular zone NSCs, and we will discuss the relevance of this important finding in terms of possible future therapeutic strategies.

## Background

Glioblastoma (GBM) is the most malignant primary brain tumor in humans. The World Health Organization classified this tumor as Grade IV glioblastoma, and consists of poorly differentiated cells with vascular proliferation and pseudopalisading necrosis. Glioblastomas are characterized by rapid cell infiltration and invasion, frequent relapses and poor prognosis, and survival rates (1). Like other cancers, GBMs show a high degree of heterogeneity in a wide range of genomic, phenotypic, and functional features (2–4). For that reason, patients with GBM exhibit a high variety of genetic abnormalities and clinical characteristics with subsequent variability in survival times and response to treatments.

Glioblastomas (GBMs) contain a small subpopulation of cancer cells with stem cell characteristics including self-renewal ability, proliferation, multilineage potency, and migration capacity, that are referred to as glioma stem cells (GSCs) (5–7). Compelling evidence from the last decade suggests that GSCs may arise from neural stem cells (NSCs) residing in the adult subventricular zone (SVZ) (8, 9). A recent article by Lee et al. (10) has provided molecular genetic confirmation of this issue showing that NSCs in the SVZ could be the cell of origin that encloses the driver mutations of human GBM. This important finding will allow the development of treatments targeting SVZ-derived NSCs harboring driver mutations.

In this review, we will focus on the existing link between NSCs in the SVZ and the initiation and development of the GBM, and we will discuss possible therapeutic interventions in the SVZ.

### Neural Stem Cells of the Subventricular Zone

**Figure 1:**
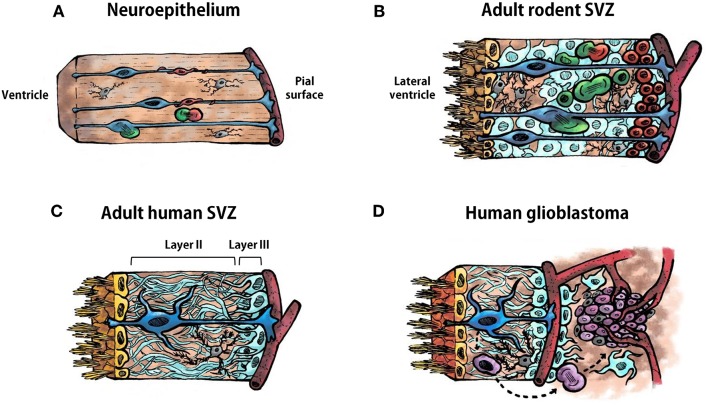
Cell types in the neuroepithelium and in the subventricular zone. **(A)** Schematic representation of the ventricular zone (neuroepithelium) during development of the vertebrate central nervous system. Radial glia are represented in blue, neural progenitor cells in green, neuroblasts in red, and microglia in gray. A blood vessel is illustrated bordering the pial surface in dark red. **(B)** Schematic representation of the adult rodent subventricular zone (SVZ). Ependymal cells are represented in yellow, type B1 astrocytes in blue, type B2 astrocytes in pale blue, type C cells (transit-amplifying neural progenitor cells) in green, type A cell (neuroblasts) in red, and microglia in gray. **(C)** Schematic representation of the human adult SVZ. Ependymal cells are represented in yellow, astrocyte-like neural stem cells in blue, astrocytes in pale blue and microglia in gray. Layer I is constituted by the ependymal cells. Layers II and III are indicated in in the illustration. Layer IV is not shown. **(D)** Schematic representation of the human adult SVZ and an adjacent glioblastoma. Ependymal cells are represented in yellow, astrocyte-like neural stem cells in blue, astrocytes in pale blue, microglia in gray, glioma stem cells in purple, other type of glioma cells in pale purple. Astrocyte-like neural stem cells acquire driver mutations that generate glioma stem cells which divide to form the tumor mass.

The adult SVZ lining the lateral ventricles contains NSCs that share features of astrocytes and of immature progenitors (11, 12). To understand the source and organization of these astrocyte-like NSCs in the adult SVZ it is necessary to revise the neurogenic process that occurs during embryonic development. The ventricular zone, or neuroepithelium, is a highly proliferative zone that expands in the early embryonic stages of development through division of neuroepithelial cells, symmetrically to expand their pool, and asymmetrically to generate differentiated progeny (13, 14). As neural tissue is added, neuroepithelial cells become elongated and extend their processes from the ventricle surface, to contact cerebrospinal fluid (CSF), to the pial surface, to contact blood vessels. These elongated neuroepithelial cells are named radial glia and are the responsible for the bulk of neurogenesis in the early embryonic brain (Figure 1A). Radial glia divide asymmetrically allowing their self-renewal and the generation of neuroblasts that migrate toward their final destination in the cortical plate with the help of the mother's radial glia extended process (Figure 1A). Microglial cells are also present in the neuroepithelium when these events, including the assembling of neural circuits, take place (Figure 1A). In addition, during the late embryonic development and the first weeks of birth, radial glia are also the source of astrocytes and oligodendrocytes, that populate the different brain structures, and of ependymal cells that will line the ventricle surface. Nowadays, it is well-assumed that astrocyte-like NSCs within the SVZ derive from embryonic radial glial cells (12, 15, 16). Specifically, in rodents, SVZ NSCs consist of a subpopulation of astrocytes (named B1 astrocytes) that differ from another population of non-neurogenic astrocytes (B2 astrocytes). B1 astrocytes are located under the layer of ependymal cells lining the ventricle and some of them have a short apical process with a single primary cilium projected toward the CSF in the lateral ventricle, and also a basal process that contacts blood vessels of the SVZ plexus (17) (Figure 1B). This strategic location allows type B1 cells to receive signals from the CSF and from the blood, as radial glia do during development. In contrast, B2 astrocytes do not contact the ventricle. Eventually, type B1 cells form transit-amplifying neural progenitor cells (type C cells) in asymmetric divisions, which, in turn, divide to give rise to neuroblasts (type A cells) (18–20) (Figure 1B). Newly-formed neuroblasts migrate in chains ensheathed by gliotubes of astrocytes toward the olfactory bulb along the rostral migratory stream (21, 22). Once in the olfactory bulb, these immature neurons differentiate into interneurons that integrate in pre-existing functional circuits (23, 24). In addition, SVZ type B cells can also generate oligodendrocyte precursors that contribute to the maintenance of the oligodendrocyte population in the neighboring corpus callosum, striatum, and fimbria-fornix both in the normal brain and after a demyelinating lesion (25–27). The SVZ is also abundant in microglial cells where they intervene in the control of postnatal and adult neurogenesis (28).

### Cytoarchitectural Distinctions of the Human Subventricular Zone

The organization of the adult human SVZ differs from the classical SVZ described above for other mammalian species. During development radial glia in the human SVZ generate neurons and macroglia that populate the developing brain. The main difference relies on the existence of an outer SVZ, which also contains radial glia from which neurogenesis takes place that leads to extensive cortical expansion. This accounts for the larger and more complex cortical characteristics of the human brain. Following corticogenesis, the neurogenic niche of the SVZ and outer SVZ remains proliferative in neonates, generating new neurons that populate the prefrontal cortex and, to a lesser extent, the olfactory bulb. After approximately 2 years, SVZ neurogenesis ceases and the SVZ acquires an organization that differs from the classical SVZ cytoarchitecture described for rodents (Figure 1C). A detailed description of the adult human SVZ was provided by Quiñones-Hinojosa et al. (29) with four layers being characterized. Layer I is composed of ependymal cells in contact with the ventricular lumen. Next to this layer, there is an almost acellular layer (Layer II), which is formed during postnatal development as a consequence of neuroblast depletion in this region. This layer contains numerous processes of astrocytes connected by junctional complexes and a few microglial cells (Figure 1C). This is probably a region of signaling exchange between astrocytes, and between astrocytes and ependymal cells. Microglia might also influence communication between these cell types. Adjacent to this hypocellular layer there is a dense ribbon of astrocytic cell bodies (Layer III) with variable morphology, whose organization resembles the glial meshwork that surrounds migrating neuroblasts in the SVZ, with the exception that in the adult human this meshwork is devoid of neuroblasts (Figure 1C). Finally, Layer IV is a transition region with few cells and similar to the underlying brain parenchyma. Some astrocytes of the adult human SVZ proliferate, as revealed by staining with Ki67 and proliferating cell nuclear antigen (PCNA) (29, 30). However, as mentioned before, neuroblasts are not found either in adult human SVZ or in the rostral migratory stream toward the olfactory bulb. Indeed, the incorporation of new neurons in the human olfactory bulb is nearly extinct by adulthood (31). Interestingly, newly generated cells in adult human brain are mainly oligodendrocytes, not neurons (31, 32), which suggests that the oligodendrogenic process and its corresponding myelin maintenance acquires more significance in the human brain compared to other mammalian brains. Therefore, NSCs remain in the SVZ of the adult human brain although their role has not yet been clearly elucidated. The investigation on key features of this population of NSCs, which constitute a substrate for neoplastic transformations, will lead us to a better understanding of neurodevelopmental, neurodegenerative, and tumorigenic pathologies.

### Cellular Constituents and Tumor Niche in Human Glioblastomas. Similarities With the Subventricular Zone Neurogenic Niche

Human GBMs consist of a heterogeneous cell population, both neoplastic and non-neoplastic, that are organized as a cellular and functional hierarchy based on a subpopulation of glioma cells with stem cell properties, the GSCs (Figure 1D). GSCs have potent tumor-initiating ability, self-renewal capacity, and resistance to standard therapies (6, 33). GSCs are the origin and source of tumor recurrence in GBM and are capable of whole tumor regeneration once the treatment has concluded (33–35). Interestingly, GSCs share common features with NSCs of the SVZ, such as nestin expression, high motility, diversity of progeny, robust proliferative potential, association with blood vessels, and bilateral communication with constituents of the niche such as endothelial cells, pericytes, astrocytes, or extracellular matrix (Table 1) (36).

**Table 1:** Common features between glioma stem cells and subventricular zone neural stem cells.

Nestin expression
Proliferative potential, motility, diversity of progeny
Association with vasculature
Bilateral crosstalk with niche components: • Endothelial cells • Pericytes • Microglia • Astrocytes • Extracellular matrix

Glioma stem cells (GSCs) of GBMs maintain localization within a vascular niche (37, 38) (Figure 1D) and display a reciprocal communication with the perivascular niche which contributes to the GBM initiation, progression, invasion, and therapeutic resistance. For instance, endothelial cells of the perivascular niche produce numerous growth factors that promote GSC self-renewal, tumorigenicity, and survival (39–41). In turn, GSCs can release cytokines and chemokines that regulate the tumor vasculature and can even transdifferentiate and generate endothelial cells or pericytes to form their own vascular niche (42–45). NSCs of the SVZ also locate in close contact to the vasculature (46, 47) and receive signals from endothelial cells and pericytes that intervene in their maintenance and proliferation (48, 49), which is not surprising since during development, germinal zone vasculature regulates neurogenesis (50). In return, NSCs also appear to have vascular cell differentiation capacities (51).

Immune cells are also important constituents of the GBM niche (Figure 1D). Microglia/macrophages are able to infiltrate in the tumor mass in response to chemoattractant cytokines released by the tumor (52–54), and this infiltration contributes to the tumor progression, since microglia acquire a tumor-promoting phenotype characterized by the release of trophic and angiogenic factors that support the tumor growth (55). In the adult SVZ, NSCs also establish important bilateral communication with microglia with relevance in the shape of neurogenesis. NSCs are able to control microglial activity and, additionally, the activation state of the microglia influences proliferation and differentiation of NSCs (28).

Astrocytes and extracellular matrix proteins of the GBM microenvironment also contribute to the support of proliferation and migration of the GSCs (56, 57) and, similarly, they intervene in the control of NSC proliferation and migration in the SVZ niche [reviewed in (58, 59), respectively].

Despite all the similarities between tumor and SVZ stem cell niches, there is a niche constituent that is specific to the SVZ, the CSF. The CSF is a source of soluble factors with a role in mediating NSC quiescence through molecules involved in G-protein-coupled receptor signaling (60).

## Frequent Gene Mutations in Human Glioblastomas

The vast majority of GBMs (80% of cases) are considered primary GBMs; they develop rapidly *de novo* in elderly patients without clinical or histological evidence of a less malignant precursor lesion. Secondary GBMs progress from low-grade gliomas such as diffuse astrocytomas or anaplastic astrocytomas and are prevalent in younger patients. Histologically, primary and secondary GBMs are indistinguishable, but they carry specific genetic alterations in cancer-driving genes (61). Typical for primary GBMs are epidermal growth factor receptor (EGFR) amplification and loss of the tumor suppressor phosphatase and tensin homolog (PTEN). Secondary GBMs are unequivocally characterized by the presence of *IDH1* (isocitrate dehydrogenase 1) mutations (62), which are absent in primary GBMs. Historically, inactivation of the tumor suppressor protein p53 gene *TP53*, has been also considered a classical feature in secondary GBMs, but infrequently in primary GBMs (61). However, more recent literature indicates that *TP53* is a key tumor suppressor for both GBM subtypes (63). Mutations in the promoter of *TERT* (telomerase reverse transcriptase) gene are often identified in GBMs (3, 64) and correlate with elevated mRNA expression and telomerase reactivation, which suggests that maintenance of the telomere is a requisite step in GBM pathogenesis (3).

Therefore, GBMs present genetic alterations in genes involved in the control of cell proliferation, apoptosis, and tissue invasion. Interestingly, with the exception of *IDH-1*, all the above mentioned genes also control these functions in NSCs from the SVZ. Next, we will describe in more detail the effect of genes that control quiescence, proliferation, differentiation, or migration of SVZ NSCs that are frequently mutated in GBMs (summarized in Table 2).

**Table 2:** Genes most frequently mutated in glioma stem cells that are involved in the control of subventricular zone neural stem cell biology.

**Genes**	**Glioma stem cells**	**Subventricular zone neural stem cells**
*TERT*	Mutations in *TERT* lead to an increase in telomerase activity	NSCs have telomerase activity derived of their *TERT* expression
*TP53*	Mutations in *TP53* lead to loss of the tumour suppressor protein p53	p53 modulates NSC proliferation and self-renewal
*PTEN*	Mutations in *PTEN* produce absence or deficiency in the tumor suppressor protein PTEN	PTEN regulates NSC migration, apoptosis and proliferation
*EGFR*	Mutations in *EGFR* produce activated EGFR signaling	EGF/EGFR signaling activates NSC proliferation
*PDGF*	Mutations in *PDGF* induce activation of the PDGF pathway	PDGF/PDGFR signaling activates NSC proliferation

*EGF, epidermal growth factor; EGFR, epidermal growth factor receptor; NSC, neural stem cell; PDGF, platelet-derived growth factor; PDGFR, platelet-derived growth factor receptor; PTEN, phosphatase and tensin homologue; TP53, tumor suppressor gene that encodes for the protein p53*.

### Telomerase

The enzyme telomerase is responsible for the maintenance of telomere length to prevent chromosomal shortening, end-to-end fusions, and apoptosis during successive rounds of cell division (65). Though expressed widely during mammalian embryogenesis and also in the prenatal brain, in adult animals telomerase expression is restricted to the SVZ and olfactory bulb, the most proliferative brain regions in rodents (66). The activity of telomerase in dividing NSCs may overcome the progressive proliferation-induced telomere shortening and promote growth and survival of adult NSCs (67).

The active telomerase enzyme consists of telomerase reverse transcriptase (TERT), telomerase RNA (TERC) and specialized proteins (e.g., dyskerin) (68). The enzyme preserves telomere stability by adding TTAGGG repeats to the end of a given chromosome in rapidly dividing cells, using its complementary TERC sequence as the template and the TERT subunit as the catalytic component. While TERC is constitutively expressed in most cells, TERT is tightly regulated and determines telomerase activity (69). TERT activity is frequently upregulated in human cancers and it is thought to be a critical mechanism that contributes to human tumorigenesis (70). Mutations in the *TERT* promoter have been detected in more than 50% of primary adult GBMs and are correlated with increased telomerase activity (3, 71). Moreover, GBM patients with *TERT* promoter mutations have lower survival times (64).

Additionally, some cancer cells use a telomerase-independent mechanism to elongate their telomeres (72). The alpha-thalassemia/mental retardation syndrome X-linked (*ATRX*) gene is a suppressor of these alternative mechanisms. Therefore, mutations in *ATXR* are also frequently identified in GBMs (73, 74). In addition, *ATXR* mutations are often associated with *IDH1* and *TP53* mutations and are also associated with poor patient prognosis (75, 76).

In line with previous work that suggested that GBM may arise from the acquisition of somatic mutations in NSCs of the SVZ (36), it is important to highlight that *TERT* promoter mutations in NSCs would permit them to develop an extended self-renewal activity, increasing their chances of acquiring GBM driver mutations over time (Figure 1D).

### Tumor Suppressor Genes

#### TP53

*TP53* is a tumor suppressor gene that encodes for the sequence-specific DNA-binding protein p53. p53 induces apoptosis or cell cycle arrest in response to genotoxic stress, thus blocking the transmission of DNA mutations to progeny cells (77). Proliferating cells of the SVZ express p53 in the embryonic and postnatal brain, where it exerts a role in the control of cell division and early differentiation rather than in the control of cell death (78). In the adult SVZ p53 also modulates proliferation and self-renewal of NSCs (79, 80). Loss of function of p53 changes the behavior of type B and type C cells leading to the formation of periventricular areas of cellular hyperplasia in the adult SVZ formed by clusters of these cell types together with neuroblasts (79). Moreover, exposition of *TP53*^−/−^ mice to the mutagen N-ethyl-N-nitrosourea (ENU) induces the formation of GBM-like tumors in the adult SVZ (81). *TP53* mutations leading to p53 loss are frequent in both GBM subtypes (61, 63).

#### PTEN

PTEN encodes a phosphatase that regulates NSC migration, apoptosis, and proliferation of mouse SVZ NSCs (82, 83). To precisely analyse the role of PTEN in human SVZ NSCs Duan et al. generated PTEN-deficient human NSCs by targeted gene editing (84) and demonstrated that PTEN deficiency induces a reprogramming of NSCs toward a GSC-like phenotype. Specifically, PTEN deficiency leads to an upregulation of PAX7, which in turn promotes oncogenic transformation of the NSCs. Patients with GBMs deficient in PTEN present increased levels of PAX7, which has been associated to the aggressive characteristics of the GSCs. Targeting PTEN-deficient NSCs emerges therefore as an important therapeutic strategy for GBMs. With that purpose, the mentioned authors used mitomycin C to selectively target NSCs with PTEN deficiency and induced their apoptosis. In a recent article by Jaraíz-Rodríguez et al., GSCs from GBM patients were targeted with a selective peptide that upregulates PTEN and as a consequence, a reduction in their survival, migration and invasion was achieved (85).

### Growth Factors

#### EGF

Epidermal growth factor (EGF) promotes proliferation of NSCs of the rodent SVZ by EGFR activation (86–88). Specifically, the majority of the EGF-responsive cells in the adult mouse SVZ are the rapidly-dividing transit-amplifying cells (type C cells) rather than the primary and less proliferative NSCs (type B1 cells). In addition, EGF prevents NSC differentiation, and EGFR signaling is associated with enhanced cellular proliferation, survival, and infiltration in the adjacent parenchyma, similar to the events observed in high-grade gliomas (88, 89). Noticeably, the EGF signaling pathway is also involved in gliomagenesis. For instance, amplification of the *EGFR* gene is a potential transformation mechanism in the development of GBM (90). What remains unsolved is which is the homolog to these type C highly EGF-responsive cells in the human SVZ. In any case, these results suggest that mutations in the *EGFR* leading to activated EGFR signaling, in more quiescent or in more proliferative NSCs of the human SVZ, may result in the migration of SVZ cells into the parenchyma and subsequent generation of gliomas or other brain tumors.

#### PDGF

Similarly to EGF, platelet-derived growth factor (PDGF) also activates proliferation of NSCs in the SVZ and creates areas of hyperplasia with features of early glioma formation (91). But contrary to EGF-responsive cells, NSCs expressing the receptor for PDGF (PDGFRα) are mainly the type B cells. PDGF stimulation blocks the ability of B cells to give rise to differentiated progeny which results in an accumulation of type C cells that invade the adjacent parenchyma. Therefore, PDFG signaling may be involved in the regulation of primary NSCs whereas EGFR signaling could rather be involved in the control of the secondary type C neural progenitors.

Activation of the PDGF pathway is also a common event in gliomagenesis and has been implicated in tumor initiation, indeed PDGF/PDGFR overexpression occurs with equal frequency in both low- and high-grade gliomas (92). In addition, *PDGF* expression in GBM correlates well with other mentioned bad prognosis factors such as *PTEN* deletion and *IDH1* mutation (93).

There are many other factors and signaling pathways involved in the control of SVZ NSC proliferation whose expression or activity is altered in GBMs. Some of these include the c-Met receptor, the transcription factor FOXO3, the Wnt pathway or the sonic hedgehog pathway (36, 94–98).

#### Neural Stem Cells of the Subventricular Zone as the Origin of Glioma Stem Cells

There is still controversy about the cell of origin of GBMs. NSCs are good candidates since they are more susceptible to malignant transformation than differentiated cells in the adult brain (9, 99). This susceptibility is derived of their ability to self-renew, proliferate, and bypass apoptosis and senescence by having the precise required cellular machinery. However, differentiated brain cells such as astrocytes, oligodendrocyte precursor cells, and neurons have also been described to be target of transformation and generate malignant gliomas (100–103).

The hypothesis that GBMs may originate from SVZ NSCs that have undergone malignant transformation has been recently demonstrated in an elegant study by Lee et al. (10). They showed that, in 56.3% of patients with IDH1 wild-type GBM, tissue from their tumor-free SVZ contained mutations in cancer-driving genes such as the mentioned *TP53, PTEN*, and *EGFR*, that were similar to those observed at high levels in the tumor. Furthermore, 42.3% of patients presented somatic mutations in the *TERT* promoter in the SVZ tissue shared with the associated tumors. In addition, they demonstrated that astrocyte-like NSCs from the SVZ carrying the driver mutations were able to migrate and develop malignant GBMs in distant brain regions. All the results demonstrate that NSCs in human SVZ tissue are the cells of origin that contain the driver mutations, at least in IDH1 wild-type GBM.

Interestingly, *TERT* promoter mutations in tumor-free SVZ tissue were identified in all patients with IDH-wild-type GBM with driver mutations. This finding suggests that *TERT* promoter mutation may be the earliest and a common genetic event by which NSCs in the SVZ, which have limited self-renewal activity, are able to avoid telomere shortening, thereby increasing the possibilities of acquiring driver mutations.

Their results, however, do not elucidate the cell of origin in IDH1-mutant GBM, which remains unknown. And still, seven out of the 16 GBM samples analyzed by Lee et al. did not have mutations in the tumor-free SVZ samples, which indicates that SVZ NSCs might not be the origin of all type of IDH1 wild-type GBMs.

In any case, their findings are the first genetic evidence on the cell of origin of GBM from human patients with this cancer. The alternative hypotheses that support the concept of dedifferentiation are based on experiments performed with rodents and no evidence has yet been provided in human GBM patients.

Lee et al. have also generated a mouse model of *p53, PTEN* and *EGFR* mutations in putative NSCs from the SVZ through genome editing (10). These mutations were selected because they are recurrent driver mutations found in tumor-free SVZ from GBM patients. Interestingly, 90% of the electroporated mice carrying these mutations developed brain tumors with the presence of the target mutations. By analyzing the progress of glioma development over time the authors showed that mutated NSCs migrated to distant brain sites and 67% of the gliomas developed in distant regions from the mutation-arising SVZ. These results indicate that NSCs harboring driver mutations migrate from the SVZ and lead to the development of malignant gliomas in distant brain regions. Interestingly enough, genome-edited NSCs with the driver mutations that migrated to the olfactory bulb differentiated into mature neurons and did not lead to gliomas. The understanding of the environmental cues existing in the olfactory bulb compared to the cues existing in other regions in which gliomas are developed (i.e., cortex) may broaden new approaches of therapy development.

#### Neural Stem Cells of the Hippocampus Have Not Been Involved in Gliomagenesis

The hippocampus of the adult mammalian brain contains NSCs that generate neurons via transit-amplifying cells, although their existence in the human brain has been subject of debate in the last few years (104, 105). Hippocampal NSCs are located in the subgranular zone, and have an apical portion with which they contact blood vessels, and a branched opposite process that contacts neuronal processes and glial cells (106, 107). In contrast to NSCs in the adult SVZ or during development, radial glia-like NSCs of the hippocampus do not contact CSF. As previously mentioned, CSF is a continuous source of soluble factors for the control of proliferation in SVZ NSCs (60) and, a failure in this control system could induce alterations in NSC biology that may increase their susceptibility to malignant transformation.

Another striking difference with the SVZ is that NSCs of the hippocampus differentiate to granule neurons in the same neurogenic niche. Therefore, the hippocampal niche favors neuronal differentiation which makes NSCs less prone to proliferation and migration and thus, less potentially tumorigenic.

The hippocampal/SVZ niches also differ in the role exerted by microglial cells in the NSC population. Microglia in the hippocampus are involved in the control of neurogenesis through phagocytosis of newborn cells that become apoptotic (108) whereas in the SVZ provide trophic support to the NSCs (109).

Interestingly, recent findings have demonstrated that the hippocampus is a region spared of GBM invasion (110). The authors suggest that the specific composition of extracellular matrix in this region may explain the lack of preference for GSC migration to this region (110). In contrast, GBM cells, from both IDH1 wild-type and mutant-type GBMs, are prone to migrate toward the SVZ and take advantage of the niche factors secreted in this region that promote proliferation and migration of progenitor cells (111, 112).

Therefore, unique features of the SVZ neurogenic niche might explain the possible oncogenic transformation of the NSCs in this niche, and not in the hippocampus, as well as the major preference of migration of GSCs to this region. As our knowledge of the neurogenic niches continues to expand, newly revealed features will also drive better understanding of tumor cause and therapy response.

## Therapeutic Implications

Glioblastomas (GBMs) are extremely difficult to treat since they are constituted by a heterogeneous group of cells with genetic and epigenetic variations, which interact with their microenvironment (blood vessels, microglia/macrophages, extracellular matrix), through different communication routes (soluble factors, gap junctions, extracellular vesicles, tunneling nanotubes), in order to support GBM progression. In addition, most chemical treatments have to deal with difficulties derived of the drug penetration through the blood-brain-barrier or of the severe side-effects.

Current treatment options for GBM include maximal surgical resection, followed by radiation and temozolomide treatment (113). Post-surgical treatments are necessary to prevent recurrence but, despite this, relapses occur and the prognosis of GBM is very poor. Even with maximal surgical resection plus radiotherapy with concomitant or subsequent chemotherapy, patients have a median overall survival rate of about 14–15 months (114). New drugs and combination therapies of radiotherapy and temozolomide together with novel radiosensitizers are continuously being tested in pre-clinical and clinical trials in order to achieve better outcomes and patients' survival (115–117), but still, compromised responses derived of the therapeutic resistance and inefficient targeting of GSCs are produced.

Tumor microenvironment is considered one of the main targets for new therapies since the dialogue established between the tumor cells and the tumor niche is essential for the tumor to progress. For instance, vascular endothelial growth factor (VEGF) is an important mediator of angiogenesis in GBM. A monoclonal antibody that inhibits VEGF signaling pathway, bevacizumab, has been used to decrease tumor angiogenesis in GBM, but it has considerable side effects (118). Tumor-associated microglia/macrophages also promote tumor progression by the release of trophic and angiogenic factors (55). It is striking that microglia modulates NSC biology in the SVZ early in development, postnatally, and later in the adulthood, but in GBMs microglia adapt a tumor-promoting phenotype. Attempts have been made to counteract microglia tumor-promoting phenotype and induce an antineoplastic phenotype. For instance, systemic administration of amphotericin B induces an increase in the immune functions of microglia and reduce the growth of the glioma-initiating cells (119). In this line, the knock-down of VEGF in myeloid cells reduces the pro-tumorigenic effects of microglia/macrophages and attenuates glioma progression (120). The extracellular matrix is also important for tumor invasion and progression. The blockade of the extracellular matrix protein laminin-411 has been shown to disrupt the perivascular GSC niche and inhibits GBM growth (121).

Another important issue to consider in terms of possible therapies is the heterogeneity of the GBM populations, with fast-dividing- and quiescent GSCs combined within the tumoral tissue (122). Conventional chemo and radiation therapies mainly target the proliferative population. Hence, targeting the quiescent GSC population, which is more resistant to therapy and can initiate tumors, in combination with existing therapies against proliferative GSCs may be critical to overcome this cancer (35, 122).

In the context of this review, we will discuss possible therapeutic options targeting GSCs, and more specifically, targeting their putative cells of origin, NSCs of the SVZ with driver mutations. Therapies aimed to develop treatments directed not only to GBM cells and their microenvironment, but also to the SVZ, must be taken into account in order to achieve better prognosis for GBM patients.

### Radiotherapy in the Subventricular Zone

Glioma stem cells (GSCs) in the human SVZ are specifically resistant to radiation *in vivo* (113). Factors released within the SVZ neurogenic niche are probably involved in this radioresistance and subsequently in potential tumor relapse. Searching treatments directed toward the blockade of the signaling mediated by these factors would improve the success of GBM radiotherapy. One of these SVZ niche factors is the chemokine CXCL12. Inhibition of CXCL12 in the SVZ promotes radiosensitization in an animal model of GBM (123) and reduces tumor cell proliferation in a GBM pre-clinical model (124). The relevance of the blockade of CXCL12 signaling in human GBMs has not yet been demonstrated.

Chen et al. showed that increasing the mean radiation dose in the SVZ after gross total resection to 40 Gy or greater, significantly improved the survival of GBM patients (125).

### Targeting Telomerase in the Subventricular Zone

As mentioned before, patients with GBM present mutations in the *TERT* promoter in the tumor-free SVZ (10). As a consequence, telomerase activity is reactivated in SVZ NSCs, providing the capacity to divide indefinitely and increasing the likelihood of mutations in oncogenic or in tumor suppressor genes. Therefore, strategies directed to target telomerase in the SVZ might be worth to be developed. Since telomerase activity is associated with a high variety of tumors, researchers have devised different methods to target telomerase as a therapeutic strategy, such as the use of TERT-specific small-molecule inhibitors, immunotherapy, gene therapy, and plant-derived compounds (70, 126, 127). Specifically, in GBM, a phase II study with i.v. administration of imetelstat, an oligonucleotide that binds to the template region of the RNA component of telomerase (TERTC), produced telomerase inhibition in the tumor and in peripheral blood mononuclear cells, but the regimen resulted too toxic in children with recurrent CNS tumors (128). Interestingly, there is a plant-derived compound that inhibits the proliferation of human GBM through the down-regulation of TERT and the consequent reduction in telomerase activity. The compound is known as butylidenephthalide, the chloroform extract of *Angelica sinensis* (129). Butylidenephthalide supresses the growth of GBM cells, *in vitro* and *in vivo* in mice injected subcutaneously with the drug (130). In order to achieve better results with this compound, a system has been designed to allow its delivery intracranially through biodegradable polyanhydride wafers (131). The authors demonstrated that the butylidenephthalide wafers reduced the size of the tumors in a dose-dependent manner without relevant adverse effects in the animals, and induced a reduction in TERT mRNA expression which leads to tumor senescence. These results represent a promising method for intervening in GBM progression and invasion. Additionally, it would be interesting to evaluate the effect of those treatments not only in the GBM tissue, but selectively targeting NSCs of the SVZ.

### Targeting Driver Mutations in the Subventricular Zone

As demonstrated by Lee et al. (10), astrocyte-like NSCs of the SVZ acquire driver mutations and are the cell of origin of GSCs that lead to GBMs. Mutated oncogenes in these SVZ NSCs could be silenced by gene editing. The discovery and application of the CRISPR/Cas9 (clustered regularly interspaced short palindromic repeats/CRISPR associate protein 9) system allows targeted and accurate genome editing, correction, and repairing (132, 133). This technology might also be used for genome correction in mutated tumor suppressor genes in SVZ NPCs that result in the development of GBMs. Recent findings by Gebler et al. (134) have demonstrated that the CRISPR/Cas9 system is sensitive enough to distinguish single base pair alteration and selectively cleavage cancer mutant genes. Furthermore, the CRISPR/Cas9 system is less genotoxic and cause less undesired DNA lesions in cells than other cancer treatment regimes employing DNA-damaging drugs and/or radiation.

However, the application of CRISPR/Cas9 technology to GBM is challenging since in this cancer type there are numerous cell clones, often with multiple mutations in different pathways per clone, making unclear which are the driver mutations to target. Further experiments and clinical trials will reveal the feasibility of this technology in GBM.

### Treatment Delivery in the Lateral Ventricles

Due to the close contact of SVZ NSCs with the lateral ventricle, a possible way to target the SVZ would be the administration of drugs, vectors or cells in the lateral ventricles. This might be an adequate approach not only to directly target the population of NSCs in the SVZ, circumventing limitations imposed by the blood brain barrier, but also to target factors in the SVZ microenvironment that might be contributing to the GBM development.

Some authors have used viral vectors encoding anti-tumor proteins infused in the lateral ventricles. For instance, Meijer et al. (135) showed that intracerebroventricular (ICV) administration of an adenovirus vector encoding interferon-beta in mice bearing GBM reduced tumor growth and improved their survival. Other authors have administered in the lateral ventricles antisense oligomers to target oncogenic small non-coding RNAs (136). This treatment, in addition to bypass the blood brain barrier, allowed a greater distribution of the oligomers in the brain than other administration routes. Cell therapy can also be achieved via ICV administration. For instance, stem cells genetically modified to release factors with antitumor effects have also been demonstrated to be efficiently administered through the ICV route (137). Transplanted cells create niches of viable cells in the SVZ from where they are able to migrate to sites of tumor infiltration. Most recently, T cells genetically modified to express chimeric antigen receptors elicited better efficacy against GBM administered by ICV infusions than by local intracranial delivery (138).

Therefore, the ICV method of delivery bypasses blood brain barrier limitation, has been shown to be effective for the delivery of molecules, viruses and cells, and due to the proximity of the SVZ, where glioma-initiating cells may be formed, emerges as a promising approach for GBM therapy that warrants further research.

## Conclusions

Shared similarities between NSCs of the SVZ and GSCs have led to the hypothesis that GBMs may arise from NSCs residing in the lining of the lateral ventricles that undergo malignant transformation.

Recent findings have corroborated this hypothesis showing that astrocyte-like NSCs of the adult human SVZ acquire driver mutations which enable them to escape from niche control leading to uncontrolled proliferation and tumorigenesis.

Mutations identified in the tumor of GBM patients and also in tumor-free SVZ tissue include low-level driver mutations in *TERT* promoter or in cancer-driving genes, such as *PTEN, TP53*, and *EGFR*.

The knowledge of the cell of origin that contain the driver mutations of GBM will allow a better understanding of the nature of the GSCs in order to overcome their resistance to chemo- and radiotherapy or to avoid their progression, and it may help in the development of novel treatment interventions for this incurable disease.

## Author Contributions

EM and AP revised the literature, designed the review and the illustrations, and wrote the manuscript.

### Conflict of Interest Statement

The authors declare that the submitted work has not been carried out in the presence of any personal, professional or financial relationships that could potentially be construed as a conflict of interest.
